# The Role of Naphthaleneacetic Acid and 1-Methylcyclopropene in Preventing Preharvest Berry Dropping in *Vitis vinifera* L.

**DOI:** 10.3390/plants14020280

**Published:** 2025-01-19

**Authors:** Antonio Carlomagno, Claudio Bonghi, Giuseppe Montanaro, Alessandra Ferrandino, Angela Rasori, Vitale Nuzzo, Vittorino Novello

**Affiliations:** 1 Department of Agricultural, Forest, Food, and Environmental Sciences, Università degli Studi della Basilicata, 85100 Potenza, Italy; giuseppe.montanaro@unibas.it (G.M.); vitale.nuzzo@unibas.it (V.N.); 2 Department of Agronomy, Food, Natural Resources, Animals and Environment, Università degli Studi di Padova, 35020 Legnaro, Italy; claudio.bonghi@unipd.it (C.B.); angela.rasori@unipd.it (A.R.); 3 Department of Agricultural, Forest and Food Sciences, Università degli Studi di Torino, 10095 Grugliasco, Italy; alessandra.ferrandino@unito.it (A.F.); vittorino.novello@unito.it (V.N.)

**Keywords:** abscission, auxin, Dolcetto, ethylene, PGRs, RNA

## Abstract

Fruit dropping represents a concern in many fruit species, including *Vitis vinifera* L. This research investigated the role of two plant growth regulators (PGRs), naphthaleneacetic acid (NAA) and 1-methylcyclopropene (1-MCP), in mitigating preharvest berry dropping (PHBD) through affecting ethylene (ET) and auxin (AUX) metabolism and interactions, key hormones involved in abscission. The experiment was carried out on cv. Dolcetto, with PGR treatments applied at 43, 53, and 90 days after anthesis (DAA) for NAA and at 56 DAA for 1-MCP. Berry dropping incidence, yield parameters, and transcript levels of genes related to ET and AUX pathways were analyzed, including *VIT_212s0059g01380*, *VIT_211s0016g02380*, *VIT_207s0005g00820*, *VIT_216s0013g00980*, *VIT_203s0091g00310*, and *VIT_207s0104g00800*. Both NAA and 1-MCP significantly reduced PHBD, with NAA achieving a 92% reduction and 1-MCP an 82% reduction compared to control vines. Transcript analysis revealed differential gene expression patterns, indicating that NAA affects the ET biosynthesis pathway, while 1-MCP interferes with ET receptor signaling. The results suggest that both PGRs effectively reduced berry dropping, providing a basis for integrated crop management strategies to mitigate PHBD in grapevine cultivars susceptible to this physiological disorder.

## 1. Introduction

Abscission is a process that leads to organ (leaf, flower, and fruit) dropping [1] at a given developmental stage [2], and it depends on many environmental and internal cues (and their interaction) [1,3,4,5]. Organ abscission implies the activation of biochemical processes [6,7] at the abscission zone (AZ) [2]. These processes include an increase in cellulase and polygalacturonase activities, promoting cell wall degradation at the AZ [8].

In tree crops, the abscission of ripe fruits just prior to being harvested (preharvest dropping) represents an issue impacting yield and in turn crop profitability, as documented, for example, in citrus [9], peach [10], and apple [11].

The activation of the AZ involves, among others, a hormonal signal triggered by ethylene (ET) [7,12] and hormonal interactions (crosstalk) between ET and auxin (AUX) [12,13]. An increase in ET usually promotes the activation of AZ, while a high AUX concentration decreases that activation [2]. Hence, the ET/AUX ratio is also involved in AZ activation and, in turn, fruit drop [14].

In horticulture, the abscission process can be inhibited (or stimulated) by using plant growth regulators (PGRs) to accomplish different purposes (e.g., advanced or delayed harvest, etc.) [15].

For example, an exogenous ET application accelerated fruit abscission in grape [16], whereas an exogenous AUX (e.g., 1-naphtaleneacetic acid, NAA) application reduced fruit abscission in apple [11]. Furthermore, considering the involvement of ET in AZ activation, other PGRs influencing the ET signal (synthesis and reception) are employed to effectively counteract fruit drop [15]. For instance, aminoethoxyvinylglycine (AVG) is an inhibitor of 1-aminocyclopropane-1-carboxylate synthase (ACS), which catalyzes 1-aminocyclopropane-1-carboxylic acid (ACC) formation, the precursor of ET. In addition, 1-methylciclopropene (1-MCP) is applied to minimize fruit drop because it is an antagonist of ET for receptor binding sites [17].

In *Vitis vinifera* L., the molecular mechanisms of organ abscission involving hormones (therefore PGRs) were documented in flower [4], fruitlet [3,18], and ripe berry during post-harvest [19], whereas limited information is available about preharvest berry dropping (PHBD).

Berry abscission susceptibility was documented to change during berry development [20], and a cultivar-to-cultivar variability exists in berry dropping sensibility [21,22]. Hence, analyzing the response of susceptible cultivar(s) [22] to PGRs might help to expand our knowledge on this specific and economically relevant topic.

In *V. vinifera* cv. Dolcetto, the AZ forms soon after veraison [23], whereas PHBD is minimal during ripening but peaks at full ripeness. The incidence of PHBD varies significantly between vintages (from 5 to 30% of potential yield) and consistently results in significant yield losses [23,24,25]. This physiological disorder remains poorly understood, with limited information available on effective management practices to mitigate it and preserve yield. Hence, Dolcetto serves as a model variety for investigating preharvest berry abscission/drop in relation to the application of PGRs.

In ripe Dolcetto berries, an anatomical study identified the AZ localized between the pedicel receptacle and the pericarp, characterized by expanded medullar parenchyma and shrinked xylem bundles [26], envisaging its potential involvement in PHBD. Reference [25] found an ET peak corresponding to the beginning of veraison when the PHBD is probably triggered [27]. Both findings pointed out a probable correlation between increasing ET at the AZ and its activation in grape berry, as shown by [18].

Within plant hormonal signaling, evidence indicates that high AUX concentrations can slow down ripening in grape berries [28] and enhance berry retention [3]. In line with this, an interplay has been reported between ET and AUX in regulating the fruitlet abscission [3,29], which would cascade from a differential expression of genes involved in ET and AUX synthesis. Therefore, it is expected that PGRs contrasting PHBD might influence the expression of ET- and AUX-related genes. However, in grapevine, this has not yet been adequately explored. Different plant species use common genes to regulate the abscission process [30]. A gene expression analysis based on those pathways involved in fruit/berry abscission [6,11,14] and berry ripening [31,32] would contribute to elucidating the process of abscission and help set management practices for limiting the dropping of grape berries, as in other fruit species [11,14,33,34].

The plant growth regulators potentially effective in preventing PHBD in *V. vinifera* cv. Dolcetto were selected by considering (a) their registration for use on grapevine or other fruit crops, (b) documented efficacy in reducing berry/fruit drop, and (c) existing studies on their impact on grape berry ripening. Both NAA and 1-MCP met at least one of these criteria.

Against this background, this study examined the effect of berry drop attenuation induced by PGRs putatively competing with ET (biosynthesis and receptors).

To test these hypotheses, a field experiment was performed on *V. vinifera* cv. “Dolcetto” sprayed with two commercially available PGRs (NAA and 1-MCP).

To complement the field observations in both the control and PGR-treated vines, the expression of the key genes putatively involved in AZ activation—and in turn PHBD—was investigated. Specifically, this study considered the transient expression of six genes encoding for (a) the ACC oxidase involved in the last step of ET biosynthesis [35,36]; (b) transcription factors leading to ET responses [37]; and (c) AUX conjugation with aspartate useful for AUX homeostasis [31,32]. The findings contribute to expanding our knowledge on PHBD in *V. vinifera* by evaluating the efficacy of the tested PGRs as potential tools for managing this disorder on a vineyard scale.

## 2. Results

### 2.1. Effects of NAA and 1-MCP on Berry Dropping

**Figure 1:**
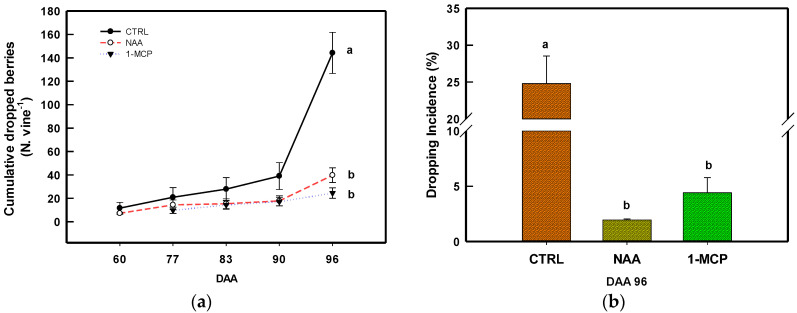
(**a**) Cumulative number of dropped berries per vine and (**b**) berry dropping incidence (% *w*/*w*) in the CTRL, NAA-, and 1-MCP-treated vines. Means ± SE bars. DAA = days after anthesis. Comparing treatments within the same DAA, different letters indicate statistically significant differences (*p* < 0.05, Student–Newman–Keuls test).

From 60 to 90 DAA, the cumulative number of dropped berries per vine soon started to increase, particularly in CTRL vines; however, no significant differences were found between treated vines (NAA and 1-MCP) and CTRL ones (Figure 1a).

During the following week, at 96 DAA, the cumulated dropping berry steeply increased up to 144.20 ± 30.38 berries vine^−1^ in the CTRL, while it was 39.72 ± 10.80 and 24.50 ± 7.65 berries vine^−1^ in NAA and 1-MCP, respectively (Figure 1a). Translating those data in terms of weight (yield) loss, the calculated *DI* at harvest for both NAA (1.95 ± 0.16% *w*/*w*) and 1-MCP (4.41 ± 1.38% *w*/*w*) vines was significantly lower than that in the CTRL, with a peak at 24.8 ± 3.74% *w*/*w* (Figure 1b).

### 2.2. Effects of NAA and 1-MCP on Transcription of Genes Involved in ET Biosynthesis and Signaling

Two genes (*VIT_212s0059g01380* and *VIT_211s0016g02380*) encoding for 1-aminocyclo-1-carboxylate oxidase (ACO) have been tested.

**Figure 2:**
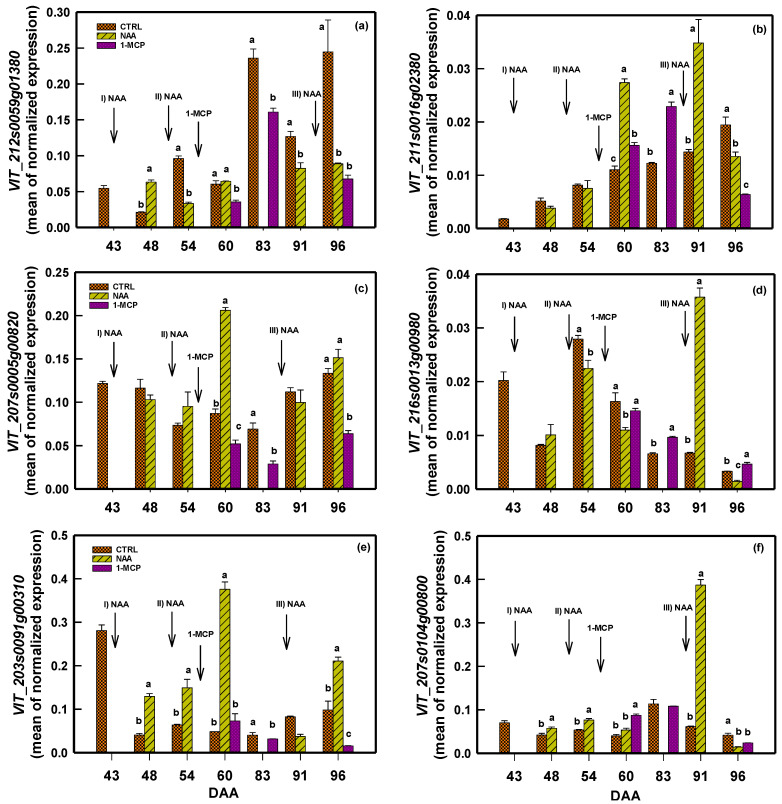
Expression of (**a**) *VIT_212s0059g01380*; (**b**) *VIT_211s0016g02380;* (**c**) VIT_207s0005g00820; (**d**) VIT_216s0013g00980; (**e**) *VIT_203s0091g00310*; and (**f**) *VIT_207s0104g00800* genes in CTRL, NAA-, and 1-MCP-treated berries. Means (n = 50) ± SE. DAA = days after anthesis. Comparing treatments within the same DAA, different letters indicate statistically significant differences (*p* < 0.05, Student–Newman–Keuls test). Arrows in each panel mark the timing of NAA (× three times at 43, 53, and 90 DAA) and 1-MCP (applied once at 56 DAA) applications.

The *VIT_212s0059g01380* transcript level in CTRL berries showed a progressive accumulation throughout ripening by reaching the highest value at harvest (Figure 2a). The NAA and 1-MCP treatments significantly affected the *VIT_212s0059g01380* expression, and it was particularly evident in the lag phase and at harvest (Figure 2a). The last NAA application effect was still evident at 96 DAA, where the expression level was significantly lower than the CTRL. At 60 DAA, 7 days after the second NAA application, the expression was not significantly different between the CTRL and NAA, whereas both were significantly higher than 1-MCP.

The transcript level of *VIT_211s0016g2380* remained unchanged after the first two NAA applications, as observing the CTRL at 48 and 54 DAA (Figure 2b). Conversely, at 60 DAA, the transcript accumulation in NAA-treated berries was significantly higher than in the CTRL. This trend was maintained also after the last NAA application, while at harvest (96 DAA), the *VIT_211s0016g02380* transcript level in NAA-treated berries sharply dropped, reaching a value lower than that in the CTRL (Figure 2b). Berries treated with 1-MCP at 60 DAA exhibited a significantly higher accumulation of *VIT_211s0016g02380* transcript compared to the CTRL group. However, this level was notably lower than that observed in NAA-treated samples. In the last two sampling points, the 1-MCP-treated berries overlapped the trend observed in NAA-treated samples (Figure 2b).

Focusing on ET responsive factors (ERF/AP2), the relative transcript content of *VIT_207s0005g00820* and *VIT_216s0013g00980* was determined. The *VIT_207s0005g00820* transcript level in NAA-treated berries peaked at 60 DAA, whereas in the other sampling dates, the transcript amount of NAA and CTRL was comparable (Figure 2c). The *VIT_207s0005g00820* transcript level in 1-MCP-treated berries form veraison to harvest was significantly lower than the CTRL (Figure 2c). *VIT_216s0013g00980* showed a relevant increase in its transcripts soon after the last NAA application (90 DAA) (Figure 2d), whereas in 1-MCP-treated berries at 83 and 96 DAA, its transcript level was higher than the CTRL berries.

### 2.3. Effect of NAA and 1-MCP on Transcription of Genes Involved in AUX Homeostasis

The relative *VIT_203s0091g00310* and *VIT_207s0104g00800* transcript levels in CTRL berries were fairly basal across the experiment (Figure 2e,f). In NAA-treated berries, the *VIT_203s0091g00310* transcript amount was significantly higher than the CTRL at 48, 54, 60, and 96 DAA. At 91 DAA, one day after the third NAA application, the *VIT_203s0091g00310* transcripts were lower than the CTRL, while for *VIT_207s0104g00800*, the highest transcript amount was registered. The 1-MCP-treated berries were characterized by a transcript amount of *VIT_203s0091g00310* similar (60 DAA) or lower (83 and 96 DAA) than that in the CTRL (Figure 2e). Considering *VIT_207s0104g00800*, instead, 1-MCP-treated berries showed a transcript amount significantly higher than the CTRL only four days after its application (Figure 2f).

### 2.4. Effects of NAA and 1-MCP on Yield and Leaf Area (LA)

Yield (Y), number of bunches per vine (n), bunch fresh weight (g), and LA per vine (m^2^ vine^−1^) were not statistically different among the treatments. Instead, the LA/Y ratio (m^2^ kg^−1^) in both NAA- and 1-MCP-treated vines resulted in being approx. 30% significantly lower than that in CTRL vines (Table 1).

### 2.5. Effects of NAA and 1-MCP on Berry Fresh Weight and Ripening

**Figure 3:**
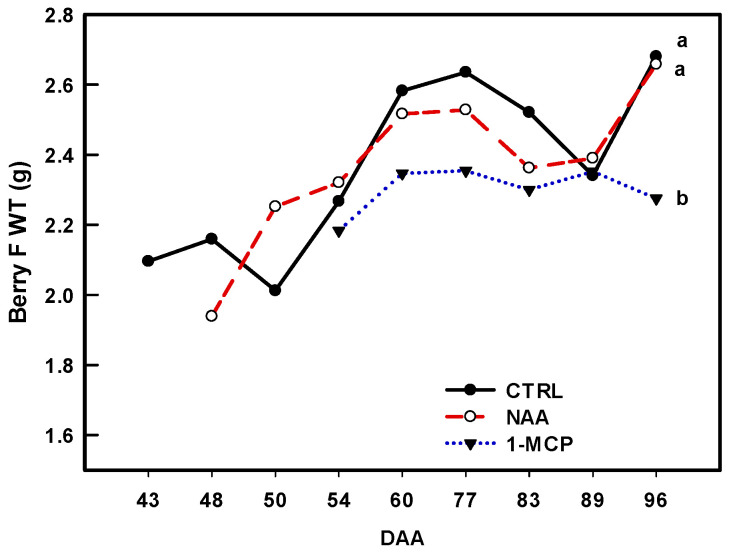
Mean values (n = 150) of berry fresh weight (F WT, g) evolution along the season in CTRL, NAA-, and 1-MCP-treated berries. DAA = days after anthesis. Comparing treatments within the same DAA, different letters indicate statistically significant differences (*p* < 0.001, Student–Newman–Keuls test).

The berry fresh weight did not show significant differences among the treatments throughout the experiment, except at harvest (96 DAA), when in 1-MCP-treated vines, it was 15% significantly lower than that in the CTRL (and NAA) (Figure 3).

**Figure 4:**
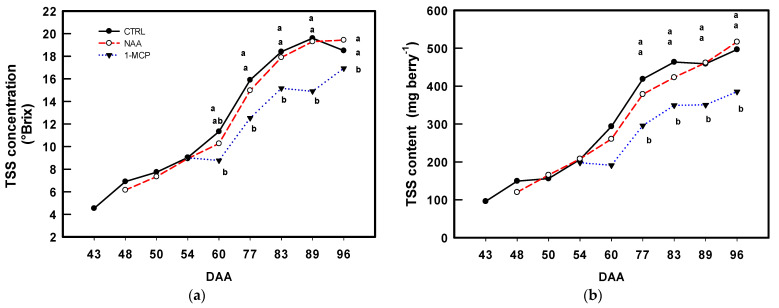
Mean values (n = 300) of total soluble solids (TSSs) (**a**) concentration (°Brix) and (**b**) content (mg berry^−1^) evolution along the season in CTRL (●), NAA- (○), and 1-MCP-treated (▼) berries. DAA = days after anthesis. Comparing treatments within the same DAA, different letters indicate statistically significant differences (*p* < 0.05, Student–Newman–Keuls test).

At 43 DAA, the CTRL berries showed 4.53 ± 0.32 °Brix (Figure 4a), hence putting them into the lag phase according to [38]. At harvest, the NAA and CTRL vines showed the significantly highest TSS values, 19.40 ± 0.40 and 18.50 ± 0.59 °Brix, respectively. By contrast, starting from 60 DAA, the 1-MCP berries showed the significantly lowest TSS concentration, and at harvest, it was 10% significantly less compared to that in the CTRL (and NAA). The NAA-treated berries did not display any significant difference in TSS content compared to the CTRL (Figure 4b), whereas in 1-MCP-treated berries, it was significantly lower than in the CTRL at 77, 83, 89, and 96 DAA (Figure 4b). Titratable acidity (TA, g L^−1^ as tartaric acid equivalents) did not show any significant differences among the treatments, except at 83 DAA, when the 1-MCP berries showed significantly higher values compared to the CTRL and NAA berries (Table S1).

## 3. Discussion

The results showed that the critical stage of berry drop occurrence is around full ripening. The application of PGRs notably diminished the incidence of berry drop measured at harvest by approximately 92% with NAA and 82% with 1-MCP of the drop recorded in CTRL vines. This result aligns with observations in other fruit species such as apple [11,14,33,34] and citrus [39], contributing to expanding knowledge on a hormone-based reduction in PHBD in grapevine.

Regarding the impact of PGRs on quality traits, only 1-MCP stood out as it significantly reduced the berry TSS content (mg berry^−1^) by approx. 24% compared to that in both the CTRL and NAA (Figure 4b), and the effect of 1-MCP on the sugar content aligns with the findings of [35] in grapevine. Considering the effect of the berry sugar content on the wine alcohol concentration, our results offer additional insights into potential strategies for addressing alcohol reduction in wine production, an emerging challenge [40]. However, this remains to be specifically tested.

### 3.1. PGRs Effects

The PGRs NAA and 1-MCP significantly controlled PHBD. However, it is worth nothing that NAA reduced PHBD following three applications, whereas 1-MCP led to a similar dropping reduction after a single application. A previous experience with one NAA application 15 days before harvest (Carlomagno, unpublished data) did not lead to a significant PHBD reduction, confirming the effectiveness of multiple NAA applications to contrast fruit drop, as reported by [33] working on apple. Moreover, one 1-MCP application at 56 DAA effectively reduced PHBD, as found in apple by [34]. These outcomes suggest that the earlier the application of PGRs, the greater the effect on fruit retention, according to [11].

The method of expressing preharvest drop in fruit crops is crucial, as it should provide an immediate understanding of yield loss. Fruit dropping is commonly quantified as either “cumulative fruit drop (%)” [11,33] or “fruit drop percentage (*w*/*w*)” [19,22]. In the present research, both metrics were adopted (Figure 1a,b). However, the calculation of DI (see Equation (3) in Materials and Methods) is to be considered an estimate, as the weight of dropped berries (numerator) corresponds to berries that had not reached full ripeness. These berries could have weighed more at harvest if they had not dropped, leading to a potential slight underestimation of DI. Nevertheless, presenting PHBD with both metrics enhances data interpretation and ensures consistency with the existing literature.

While NAA and 1-MCP demonstrated similar effectiveness on reducing berry drop, they exhibited different impacts on berry growth and TSSs. At the last sampling point, 1-MCP berries showed significantly ~15% lower berry fresh weight than the CTRL and NAA, in agreement with the results in [41].

The berry fresh weight increased from 43 to 77 DAA across all treatments, although this increase was less pronounced in 1-MCP-treated vines. Later on, the fresh weight displayed a notable decline in both the CTRL and NAA samples until 89 DAA. Although post-veraison berries are less sensitive to vine water status [42,43], the lack of precipitation from 71 to 88 DAA (Table S2) coupled with berry transpiration [44] might have contributed to a temporary berry shrivel. Berry fresh weight resumption was observed in NAA and the CTRL at 96 DAA but not in 1-MCP berries (Figure 3), showing the involvement of PGRs in berry growth/hydration dynamics [28]. The behavior of NAA and CTRL berries was likely due to the 53 mm of rain from 89 to 95 DAA (Table S2) that might have triggered ‘berry mass growth’ via its hydration. Post-veraison grape berry’s connection to the mother plant is extensively discussed [45] in the literature. However, the berry mass increase observed in NAA and the CTRL was probably supported by the phloem sap before becoming impeded [46]. 1-MCP had an adverse impact on both berry growth (Figure 3) and sugar content (Figure 4b), arguably interfering with the phloem unloading process (see later). Finally, to explain berry regrowth in NAA-treated vines, we evoke the positive effect of multiple NAA applications increasing ET synthesis [34] and ET function in fruit expansion [47], indirectly supported by the transient increase in the *VIT_211s0016g02380* transcript level (Figure 2b). Conversely, 1-MCP likely inhibited both ET biosynthesis by blocking its receptors [17], vanishing any potential rain-induced berry regrowth via cell expansion.

In line with the 1-MCP effects in retarding fruit ripening in berry [35], apple [14,34], and fig [41], 1-MCP-treated berries displayed lower TSSs than the CTRL and NAA throughout ripening (Figure 4a,b), indirectly suggesting the involvement of ET in sugar transport [48].

Titratable acidity (TA) was significantly affected by 1-MCP only at 83 DAA (Table S1), with values approximately 30% higher than those of the CTRL and NAA-treated berries. This transient effect of 1-MCP on TA suggests that (a) ET likely plays a role in the degradation of organic acids (mainly malic acid) [48], and (b) the effect of 1-MCP on organic acid degradation is temporary, as no differences in TA were observed among the treatments at 89 and 96 DAA.

Given the importance of TA reduction during the progression of berry ripening, these results highlight the delaying effect of 1-MCP on the ripening process, as observed in apple fruit [49]. In this experiment, all grapes were harvested simultaneously at 96 DAA to standardize comparisons among the treatments, which did not allow for assessing whether extending the ripening period for 1-MCP-treated berries might have enabled them to reach comparable TSS levels to the CTRL and NAA-treated berries.

However, based on the literature findings, it seems more plausible that 1-MCP induces a delay in ripening rather than inhibiting it altogether [50,51]. This aligns with the fact that grape berries are non-climacteric fruits, where ET is not the primary driver of ripening but is involved in a crosstalk with other hormones [48,52]. Lastly, the ripening-delaying effect of 1-MCP in grape berries only becomes evident when it is applied during the endogenous ET peak [35].

The interplay between PGRs and phloem flow, involving both berry hydration and the accumulation of solutes, as well as the 1-MCP metabolism during non-climacteric fruit ripening, opens up future research.

According to the literature [53], the optimum leaf area surface to ripe 1 kg of grape (LA/Y ratio, m^2^ kg^−1^) is ≈1.0–1.5 m^2^. Both the NAA and 1-MCP vines showed values close to that ratio, whereas the CTRL vines displayed a significant 1.4 higher LA/Y ratio compared to the treatments, denoting an apparent imbalance of the ratio. This impairment was clearly due to the higher *DI* that affected the CTRL compared to the NAA and 1-MCP ones (Figure 1). However, the differences in the LA/Y ratio among treatments likely had no significant influence on berry quality [54,55]. Consequently, the observed effects on berry quality (TSS and TA) in this study can be attributed to the application of PGRs.

### 3.2. PGRs and PHBD Interaction Through Gene Expression

This study did not consider measuring the ET and AUX during ripening; however, the existing literature indicates a consensus that the ET production peaks around veraison in several *V. vinifera* cultivars, including Dolcetto [25,35], while the AUX content is decreasing after the veraison [31].

Two genes (*VIT_212s0059g01380* and *VIT_211s0016g02380* ) encoding for ACO have been tested by considering their expression profile during berry grape development [56] and in response to berry nutritional status [57]. The ACO family genes link their expression with ET biosynthesis [48]. In the present study, *VIT_212s0059g01380* and *VIT_211s0016g02380* were used to infer PGRs’ action towards ET and in turn on PHBD, displaying contrasting results. Indeed, the application of NAA during pre-veraison induces the transient accumulation of the *VIT_212s0059g01380* transcript. This observation confirms the result reported for *VIT_212s0059g01380* by [31] in grape berries (cv. Merlot) when treated with NAA. A similar effect on an ACO gene was also noted in cherries when treated with NAA at the straw-color phase (the inception of veraison) [58]. This behavior likely arises from the necessity to maintain a correct interplay between AUX and ET, which plays a crucial role in initiating the ripening process of non-climacteric fruit [59]. This view is also consistent with the induction in the pre-veraison stage of the *VIT_203s0091g00310* and *VIT_207s0104g00800* (Figure 2e,f), two GH3 encoding for GH3 AUX conjugate enzymes involved in AUX homeostasis [3]. The control of the AUX content is essential for the correct progression of berry development and ripening [31], and in fruit drop [60]. Hence, *VIT_203s0091g00310* and *VIT_207s0104g00800*, encoding for GH3 AUX conjugate, were analyzed for their role in AUX↔ET crosstalk during grape berry abscission [3]. The increase in GH3 transcripts following the NAA treatment has been observed in cv Merlot grape berries [31] and in strawberries [61]. Conversely, both NAA and 1-MCP downregulated *VIT_212s0059g01380* in the late developmental stage (Figure 2a), envisaging an ET reduction within the berries [35,48], translating in the reduction in PHBD (Figure 1a,b). Indeed, at harvest, the accumulation of *VIT_212s0059g01380* transcripts is significantly lower in NAA and 1-MCP in comparison to CRTL (Figure 2a). During ripening, the *VIT_211s0016g02380* expression pattern was modulated in an opposite manner by NAA in comparison to *VIT_212s0059g01380* (Figure 2b). The positive effect of NAA on the accumulation of transcripts encoding ACO genes during the ripening of non-climacteric fruits has also been observed in strawberries [62]. The profile of *VIT_211s0016g02380* transcript accumulation is indicative of an AUX-induced activation of biosynthesis and a subsequent homeostatic response restoring normal ET levels for ripening non-climacteric fruits. On the other hand, the ability of NAA in preventing apple preharvest drop seems not to be coherent with the expression profile of the ACO gene (*MdACO1*), which is induced by NAA [11]. It seems more probable that NAA operates through the alteration of the ET perception/signaling pathway, as demonstrated by the strong effect of the mixture NAA/1-MCP in preharvest drop reduction [33]. This information can help to discuss the result of the 1-MCP application in this research. Indeed, the use of 1-MCP action towards ACO genes seems erratic (Figure 2a,b), while genes involved in ET signaling, as *VIT_207s0005g00820* and *VIT_216s0013g00980*, are clearly downregulated (Figure 2c,d), hence impairing the role of ET in triggering berry abscission. Furthermore, the downregulation of these two ERF/AP2 genes significantly impacts the accumulation of ET-dependent gene transcripts, which in grapevine have been suggested to limit berry expansion and sugar accumulation [31]. In line with this, a reduction in berry growth and TSSs was observed in berries treated with 1-MCP (Figure 3 and Figure 4).

Considering that NAA and 1-MCP are both able to reduce preharvest berry drop (Figure 1) and their different impacts on grape berry fresh weight (Figure 3) and TSSs (Figure 4a,b), it seems that NAA hindered the abscission/dropping by impairing AUX↔ET crosstalk and consequently the normal progression of ripening and senescence [3,29,31]. In contrast with this, the ability of 1-MCP to reduce berry drop seems more related to a weaker ET transduction signal associable to abscission, as observed in apples [34].

## 4. Materials and Methods

### 4.1. Plant Materials

The experiment was carried out in Piedmont (Northwest Italy) (Monforte d’Alba, 44°35′38″ N, 7°57′38″ E) in 2014 at a vineyard of the *V. vinifera* L. cv. Dolcetto in a hilly landscape and soil. Dolcetto vines (clone CVT CN 22) were grafted onto *V. berlandieri* × *V. riparia* 420A rootstock, planted at 2.50 × 1.00 m distance (4000 vines/ha). The vineyard soil was clay–loam; vines were rain-fed and the vineyard was southeast-exposed, with north–south row orientation. The vines were vertically shoot positioned (VSP) trained and pruned according to the Guyot system. The vineyard canopy was uniformly managed by means of shoot positioning, apical topping, and leaf-plucking. During the experimental season, locally conventional agronomic and phytosanitary practices were applied in the vineyard.

The 2014 vegetative season, from 1st of April to 30th of September, was characterized by (a) a mean air temperature equal to 19.2 °C; (b) a rainfall equal to 511 mm with 39 rainy days; and (c) a Huglin Index equal to 2476. Detailed precipitation, air temperature, relative humidity, and vapor pressure deficit data from 0 to 96 days after anthesis (DAA) are provided in the Supplementary Materials (Table S2) (data source: Regione Piemonte Settore Fitosanitario—Sez. Agrometeorologica).

### 4.2. Experimental Design and Treatments Application

**Figure 5:**
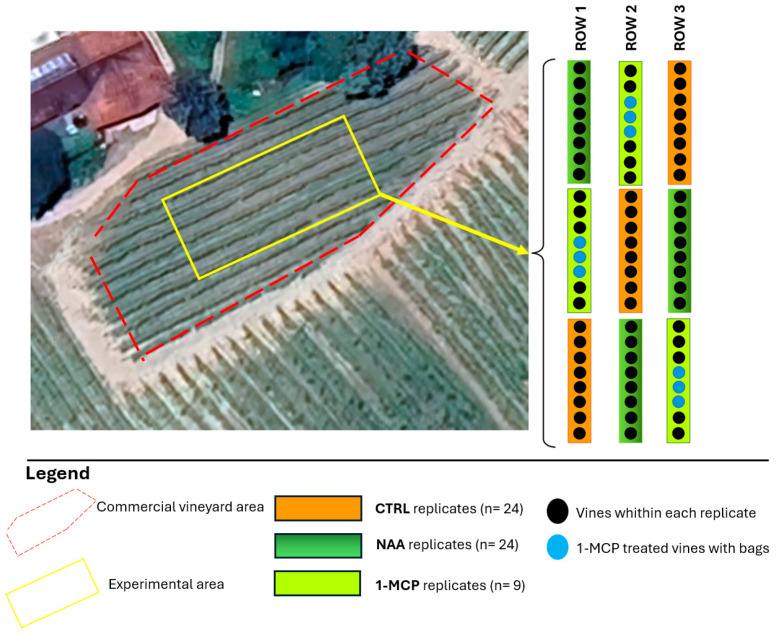
Experimental design.

The experiment was designed with two treatments (NAA and 1-MCP) and an untreated control (CTRL). A block of 57 homogenous vines was selected within the vineyard and organized with a triplicated design. A group of eight contiguous vines represented the experimental unit in both NAA and the CTRL and it was randomly replicated three times. A group of three contiguous vines (×3 reps) represented the experimental unit in 1-MCP. The treatment distribution is outlined in Figure 5.

#### 4.2.1. Naphthaleneacetic Acid (NAA)

The three field replicates (n = 24 vines) were sprayed with a 45 mg L^−1^ aqueous solution of NAA (Obsthormon 24a^®^, NAA, 7.5% *w*/*w*; L. Gobbi, Campo ligure, GE, Italy) and labeled as NAA. The NAA solution was applied by a backpack-sprayer to the whole canopy until run-off at three dates corresponding to 43 (lag phase), 53 (beginning of veraison), and 90 (preharvest) DAA. The NAA concentration to apply was chosen arbitrarily but considering (a) concentrations reported in similar research on other fruit crops, (b) label recommendations for commercially available NAA permitted in viticulture, and (c) the impact of high NAA concentrations on berry ripening [31]. The NAA timing was previously discussed (see Section 3.1).

#### 4.2.2. 1-Methylciclopropene (1-MCP)

An additional three replicates (n = 9) were selected for the 1-methylciclopropene application and 45 bunch-bearing shoots labeled as 1-MCP. 1-MCP was applied at full veraison (56 DAA) when the ET peak was supposed to occur [25,35]. Due to its gaseous state, the 1-MCP application was arranged as hereafter described.

In each replicate, 15 bunch-bearing shoots of three consecutive vines were wrapped in a polyethylene bag (bag volume equal to 0.07 m^3^); 2 shoots were enclosed in each bag. 1-MCP was obtained by weighing 1 g of SmartFresh™ 0.14 VP (a.i. 3.3% *w*/*w*; Agrofresh Inc., Rohm and Haas, Spring House, PA, USA). The powder was transferred into a plastic bottle, 100 mL of distilled water was added, and the bottle was immediately closed with a screw cap and placed inside the bag. The bag was sealed to ensure shoot isolation and preventing 1-MCP loss, and by hand, the screw cap was removed, ensuring that 1-MCP was released (4.72 ppm within the plastic bag volume) through the reaction of SmartFresh™ powder with water. The bag was shaded with a shadow net to avoid shoot/bunch overheating and removed after 24 h. The 1-MCP concentration and reacting time were adapted from [35].

### 4.3. Monitoring of Phenological Stages

During the season, the occurrence of the main phenological stages (anthesis, veraison, and harvest; Table 2) was recorded according to BBCH-identification keys (adapted for grapevine by [63]). To assess BBCH stage, thirty bunches per each replicate (see the next section) were observed as follows: ten bunches on the east side, ten bunches in the inner part, and ten bunches on the west side of the canopy. Treatment and date were expressed as days after anthesis (DAA). Anthesis was established at roughly 50% of cap-fall. Table 2 provides information about the phenological stage occurrence during the growing season, giving meaning to the calculated DAA.

### 4.4. Leaf Area (LA) Assessment

At 90 DAA, the LA of the individual vines was appraised (n = 24 in NAA and CTRL; n = 9 in 1-MCP) according to the inclined point quadrat method, as reported in [64]. Briefly, a 1.60 m. long and straight woody rod was crosswise inserted through the canopy, and the number of ‘contact’ between the rod and leaves was recorded at three different heights along the vertical component of the canopy (top, medium, and bottom part). The average number of foliar contacts (between top, medium, and bottom) corresponds to the number of leaf layers measured per vine. The LA per vine was then calculated as follows:

LA = (Pd × h) × ll  [m^2^ vine^−1^] (1)

where *Pd* represents the distance (m) between two vines along the row, *h* (m) is the height of the whole canopy, and *ll* (n) is the number of leaf layers.

According to [46], the LA to yield per vine (Y) ratio (m^2^ kg^−1^) was also calculated.

### 4.5. Berry Growth and Quality Traits

A sample of 300 berries was collected from each replicate of NAA, 1-MCP, and CTRL vines. Berries were collected from both canopy sides and detached from the rachis in small groups of 3 to 5 each from the upper, middle, and bottom parts of each cluster (roughly 60 clusters distributed in the 8 vines of each replicate in NAA and CTRL; roughly 30 clusters distributed in the 3 vines of each replicate in 1-MCP). Berries were stored in a portable refrigerator and transported to the laboratory for analysis. A subgroup of 50 berries per each replicate was used for the mean berry weight determination by measuring one by one a singular berry through a precision scale (0.001 g; Kern PLS—Kern & Sohn, Balingen, Germany). A subgroup of 100 berries per each replicate was crushed to determine the must total soluble solid concentrations (TSS, °Brix) using a digital refractometer (ATAGO, PR-32—ATAGO, Italy), and the titratable acidity (TA, g L^−1^ as tartaric acid equivalents) was assessed using the method reported in OIV (Compendium of International Methods of Wine and Must Analysis. Red, 2, 0-0097).

The TSS content per berry was calculated according to the following equation:

(2) $$TSS\;content=\left(\frac{TSS\times berry\;weight}{100}\right)\times 1000\;\left[mg\;{berry}^{-1}\right]$$

### 4.6. Berry Dropping Assessment

In all the NAA, 1-MCP, and CTRL replicates, a net was positioned on the ground under the vines catching both sides of the canopy. Starting from 60 DAA until harvest (96 DAA), all the berries dropped in each replicate were picked, counted, and weighed weekly. The cumulative dropped berries per vine were calculated by summing the numbers of berries shed at each sampling point. Data are reported as an average number of dropped fruit per vine. At harvest, the dropping incidence (*DI*, % weight/weight) was calculated as the ratio between the mass of the total dropped berries (g per replicate; *Dw*) and that of yield (g per replicate; assessed at harvest):

(3) $$DI=\left(\frac{Dw}{Yield}\right)\times 100\;\left[\%\;w/w\right]$$

### 4.7. RNA Extraction and Transcript Analysis via q-RTPCR

Gene expression analysis was performed on 50 berries per replicate sampled at 43, 48, 54, 60, 83, 91, and 96 DAA and immediately frozen in liquid nitrogen, carried in the laboratory, and stored at −80 °C until extraction.

RNA extraction and real-time PCR analysis were performed as described by [31] by using specific primers of the selected genes (*VIT_212s0059g01380*, *Vv*ACO; *VIT_211s0016g02380*, *Vv*ACO1; *VIT_207s0005g00820*, *Vv*ERF1; *VIT_216s0013g00980*, *Vv*ERF4; *VIT_203s0091g00310*, *Vv*GH3-1; and *VIT_207s0104g00800*, *Vv*GH3-8) (Supplementary Table S3). Gene expression values were normalized to the housekeeping gene *VvUbiCF* (Ubiquitin Conjugating Factor; *VIT_219s0015g01190*; [65]) and reported as arbitrary units of the mean of normalized expression using Equation (2) of Q-Gene [66].

### 4.8. Data Analysis

Within the same treatment, the data gained from the three replicates were averaged and the ± standard error (SE) was calculated. To assess the differences between the means, a one-way ANOVA was run, followed by the Student–Newman–Keuls test as a post hoc test, and *p* values lower than 0.05 were considered significant. The ANOVA assumptions were checked using the Shapiro–Wilk (normality) and Levene’s (equal variance) tests. In case of the failure of the tests, the Kruskal–Wallis test was used. All statistical analysis and charts were obtained by using SigmaPlot 12.3 (Systat Software Inc., San José, CA, USA).

## 5. Conclusions

The application of PGRs (NAA, 1-MCP) was effective in reducing berry drop (approx.—92% and—82% in NAA and 1-MCP, respectively) in Dolcetto, a grapevine cultivar very susceptible to PHBD. This would translate in saving about 1.7 t ha^−1^ yield, contributing to crop profitability. Considering the relevance of PHBD in grape cropping and the unsuitability of these cultivars for mechanical harvesting, these findings create the base for the integration of crop management aimed at reducing berry drop incidence, being beneficial for the whole viticulture industry. The transcript analysis allows for the conclusion that both the PGRs used might have influenced the level of plant hormones in grape berries, making them less prone to abscission. Hence, although more anatomical data are desirable, it appears that the PGR-induced reduction in berry drop incidence reported in this study is well grounded on molecular processes, reinforcing the replicability of the results. Considering that a sprayable commercial formulation of 1-MCP permitted also in grape is not yet available, and that 1-MCP was supplied via a time-consuming protocol (i.e., wrapping the vines), the use of 1-MCP appears to be poorly scalable to the real world. Therefore, it might be concluded that NAA (already permitted in grapevine) is a promising PGR controlling berry drop in PHBD-susceptible cultivars. However, while NAA is widely used in table grape cropping (e.g., bunch stretching, berry sizing, etc.), its application in wine grape cultivation remains less common, opening further research up to explore its potential. Finally, considering that the berry drop incidence in cv. Dolcetto has been reported to range from 5 to 30%, the dropping incidence observed in CTRL vines in the present study reinforces the reliability of the results on berry drop management.

## Figures and Tables

**Table 1:** Vegetative and productive traits measured at harvest (96 DAA) in control (CTRL), NAA-, and 1-MCP treated Dolcetto vines.

Treatment	Bunches	Bunch Fresh Weight	Yield	Leaf Area	Leaf Area/Yield
Units	(n vine^−1^)	(g)	(kg vine^−1^)	(m^2^ vine^−1^)	(m^2^ kg^−1^)
CTRL	5.97 ± 0.20	229.17 ± 23.78	1.38 ± 0.18	2.83 ± 0.17	2.06 a ± 0.05
NAA	6.78 ± 0.72	281.53 ± 2.89	1.91 ± 0.19	2.63 ± 0.16	1.39 b ± 0.11
1-MCP	6.27 ± 0.58	281.67 ± 9.28	1.69 ± 0.06	2.42 ± 0.17	1.43 b ± 0.03

Data are expressed as means ± SE (n = 24 in NAA and CTRL; n = 9 in 1-MCP). Different letters indicate statistically significant differences (*p* < 0.05, Student–Newman–Keuls test); note that letters were not reported in the case of non-significant differences.

**Table 2:** Occurrence of phenological stages and corresponding BBCH code and day after anthesis (DAA) recorded during the experiment.

Phenological Stage	BBCH Stage	Date	DAA
Anthesis	65	12 June	0
Veraison	81	30 July	48
Harvest	89	16 September	96

## Data Availability

The raw data supporting the conclusions of this article will be made available by the authors on request.

## Supplementary Materials

The following supporting information can be downloaded at: https://www.mdpi.com/article/10.3390/plants14020280/s1.

## References

[1] Li J., Su S. (2024). Abscission in plants: From mechanism to applications. Adv. Biotechnol..

[2] Olsson V., Butenko M.A. (2018). Abscission in plants. Curr. Biol..

[3] Kühn N., Abello C., Godoy F., Delrot S., Arce-Johnson P. (2014). Differential behavior within a grapevine cluster: Decreased ethylene-related gene expression dependent on auxin transport is correlated with low abscission of first developed berries. PLoS ONE.

[4] Domingos S., Fino J., Cardoso V., Sánchez C., Ramalho J.C., Larcher R., Paulo O.S., Oliveira C.M., Goulao L.F. (2016). Shared and divergent pathways for flower abscission are triggered by gibberellic acid and carbon starvation in seedless *Vitis vinifera* L.. BMC Plant Biol..

[5] Patharkar O.R., Walker J.C. (2018). Advances in abscission signaling. J. Exp. Bot..

[6] Bonghi C., Tonutti P., Ramina A. (2000). Biochemical and molecular aspects of fruitlet abscission. Plant Growth Regul..

[7] Botton A., Ruperti B. (2019). The yes and no of the ethylene involvement in abscission. Plants.

[8] Deng Y., Wu Y., Li Y., Yang M., Shi C., Zheng C. (2007). Studies of postharvest berry abscission of ‘Kyoho’table grapes during cold storage and high oxygen atmospheres. Postharvest Boil. Technol..

[9] Spiegel-Roy P., Goldschmidt E.E. (1996). The Biology of Citrus.

[10] Zanchin A., Marcato C., Trainotti L., Casadoro G., Rascio N. (1995). Characterization of abscission zones in the flowers and fruits of peach *Prunus persica* L. Batsch. New Phytol..

[11] Dal Cin V., Danesin M., Botton A., Boschetti A., Dorigoni A., Ramina A. (2008). Ethylene and preharvest drop: The effect of AVG and NAA on fruit abscission in apple *Malus domestica* L. Borkh. Plant Growth Regul..

[12] Brown K.M. (2006). Ethylene and abscission. Physiol. Plant..

[13] Taylor J.E., Whitelaw C.A. (2001). Signals in abscission. New Phytol..

[14] Li J., Yuan R. (2008). NAA and ethylene regulate expression of genes related to ethylene biosynthesis, perception, and cell wall degradation during fruit abscission and ripening in ‘Delicious’ apples. J. Plant Growth Regul..

[15] Ordoñez Trejo E., Brizzolara S., Cardillo V., Ruperti B., Bonghi C., Tonutti P. (2023). The impact of PGRs applied in the field on the postharvest behavior of fruit crops. Sci. Hortic..

[16] Ferrara G., Mazzeo A., Matarrese A.M., Pacucci C., Trani A., Fidelibus M.W., Gambacorta G. (2016). Ethephon as a potential abscission agent for table grapes: Effects on pre-harvest abscission, fruit quality, and residue. Front. Plant Sci..

[17] Blankenship S.M., Dole J.M. (2003). 1-Methylcyclopropene: A review. Postharvest Biol. Technol..

[18] Hilt C., Bessis R. (2003). Abscission of grapevine fruitlets in relation to ethylene biosynthesis. VITIS.

[19] García-Rojas M., Meneses M., Oviedo K., Carrasco C., Defilippi B., González-Agüero M., León G., Hinrichsen P. (2018). Exogenous gibberellic acid application induces the overexpression of key genes for pedicel lignification and an increase in berry drop in table grape. Plant Physiol. Biochem..

[20] Burger D.A., Jacobs G., Huysamer M., Taylor M.A. (2005). Berry abscission in *Vitis vinifera* L. cv. Waltham Cross: Changes in abscission-related factors during berry development. S. Afr. J. Enol. Vitic..

[21] Silva R.S., Silva S.M., Rocha A., Dantas R.L., Schunemann A.P.P., Pereira W.E. Influence of 1-MCP on Berry Drop and Quality of Isabel Grape. Proceedings of the VII International Postharvest Symposium.

[22] Zhu M., Zheng L., Zeng Y., Yu J. (2022). Susceptibility of two grape varieties to berry abscission. Sci. Hortic..

[23] Schneider A., Montacchini F. (1978). Aspetti morfologici e istologici della zona di ascissione nei frutti di *Vitis vinifera* L.. Allionia.

[24] Botta R., Vallania R., Me G., Luzzati A., Siragusa N. Investigation on Factors Affecting Early Dropping in Dolcetto *Vitis vinifera* L.. Proceedings of the International Symposium on Quality of Fruit and Vegetables: Influence of Pre- and Post-Harvest Factors and Technology.

[25] Gangemi L. (2005). Pre-Harvest Berry Dropping in *Vitis vinifera* L. cv ‘Dolcetto’ N. Ph.D. Thesis.

[26] Schneider A., Gay G. (1979). Mechanical harvesting and abscission of the berries in grapevine grown for wine. Ann. Della Fac. Di Sci. Agrar. Della Univ. Degli Studi Di Torino.

[27] Zhao M., Shi C.L., Li J. (2024). Abscission cues generated within the abscising organ and perceived by the abscission zone in woody fruit crops. Fruit Res..

[28] Davies C., Böttcher C., Nicholson E.L., Burbidge C.A., Boss P.K. (2022). Timing of auxin treatment affects grape berry growth, ripening timing and the synchronicity of sugar accumulation. Aust. J. Grape Wine Res..

[29] Costantini E., Landi L., Silvestroni O., Pandolfini T., Spena A., Mezzetti B. (2007). Auxin synthesis-encoding transgene enhances grape fecundity. Plant Physiol..

[30] Merelo P., Agustí J., Arbona V., Costa M.L., Estornell L.H., Gómez-Cadenas A., Coimbra S., Gómez M.D., Pérez-Amador M.A., Domingo C. (2017). Cell wall remodeling in abscission zone cells during ethylene-promoted fruit abscission in citrus. Front. Plant Sci..

[31] Ziliotto F., Corso M., Rizzini F.M., Rasori A., Botton A., Bonghi C. (2012). Grape berry ripening delay induced by a pre-véraison NAA treatment is paralleled by a shift in the expression pattern of auxin-and ethylene-related genes. BMC Plant Biol..

[32] Böttcher C., Boss P.K., Davies C. (2011). Acyl substrate preferences of an IAA-amido synthetase account for variations in grape *Vitis vinifera* L. berry ripening caused by different auxinic compounds indicating the importance of auxin conjugation in plant development. J. Exp. Bot..

[33] Yuan R., Carbaugh D.H. (2007). Effects of NAA, AVG, and 1-MCP on ethylene biosynthesis, preharvest fruit drop, fruit maturity, and quality of ‘Golden Supreme’ and ‘Golden Delicious’ apples. HortScience.

[34] Yuan R., Li J. (2008). Effect of sprayable 1-MCP, AVG, and NAA on ethylene biosynthesis, preharvest fruit drop, fruit maturity, and quality of ‘Delicious’ apples. HortScience.

[35] Chervin C., El-Kereamy A., Roustan J.P., Latché A., Lamon J., Bouzayen M. (2004). Ethylene seems required for the berry development and ripening in grape, a non-climacteric fruit. Plant Sci..

[36] Wang K.L.C., Li H., Ecker J.R. (2002). Ethylene biosynthesis and signalling networks. Plant Cell.

[37] Binder B.M. (2002). Ethylene signaling in plants. J. Biol. Chem..

[38] Coombe B.G., McCarthy M.G. (2000). Dynamics of grape berry growth and physiology of ripening. Aust. J. Grape Wine Res..

[39] Nartvaranant P. (2018). The influence of exogenously applied 2, 4-D and NAA on fruit drop reduction in pummelo cv. Thong Dee. Int. J. Fruit Sci..

[40] Varela C., Dry P.R., Kutyna D.R., Francis I.L., Henschke P.A., Curtin C.D., Chambers P.J. (2015). Strategies for reducing alcohol concentration in wine. Aust. J. Grape Wine Res..

[41] Freiman Z.E., Rodov V., Yablovitz Z., Horev B., Flaishman M.A. (2012). Preharvest application of 1-methylcyclopropene inhibits ripening and improves keeping quality of ‘Brown Turkey’ figs *Ficus carica* L.. Sci. Hortic..

[42] Hunter J.J., Volschenk C.G., Novello V., Strever A.E., Fouché G.W. (2014). Integrative effects of vine water relations and grape ripeness level of *Vitis vinifera* L. cv. Shiraz/Richter 99. I. Physiological changes and vegetative-reproductive growth balances. S. Afr. J. Enol. Vitic..

[43] Knipfer T., Wilson N., Jorgensen-Bambach N.E., McElrone A.J., Bartlett M.K., Castellarin S.D. (2023). Cessation of berry growth coincides with leaf complete stomatal closure at pre-veraison for grapevine *Vitis vinifera* subjected to progressive drought stress. Ann. Bot..

[44] Greenspan M.D., Schultz H.R., Matthews M.A. (1996). Field evaluation of water transport in grape berries during water deficits. Physiol. Plant..

[45] Carlomagno A., Novello V., Ferrandino A., Genre A., Lovisolo C., Hunter J.J. (2018). Pre-harvest berry shrinkage in cv ‘Shiraz’ *Vitis vinifera* L.: Understanding sap flow by means of tracing. Sci. Hort..

[46] McCarthy M.G., Coombe B.G. (1999). Is weight loss in ripening grape berries cv. Shiraz caused by impeded phloem transport?. Aust. J. Grape Wine Res..

[47] Huang W., Tan C., Guo H. (2024). Ethylene in fruits: Beyond ripening control. Hortic. Res..

[48] Wang P., Yu A., Ji X., Mu Q., Haider M.S., Wei R., Leng X., Fang J. (2022). Transcriptome and Metabolite Integrated Analysis Reveals That Exogenous Ethylene Controls Berry Ripening Processes in Grapevine. Food Res. Int..

[49] Doerflinger F.C., Al Shoffe Y., Sutanto G., Nock J.F., Watkins C.B. (2024). Preharvest 1-methylcyclopropene (1-MCP) treatment effects on quality of spot and strip picked ‘Gala’ apples at harvest and after storage as affected by postharvest 1-MCP and temperature conditioning treatments. Sci. Hortic..

[50] Zhang J., Ma Y., Dong C., Terry L.A., Watkins C.B., Yu Z., Cheng Z.M.M. (2020). Meta-analysis of the effects of 1-methylcyclopropene (1-MCP) treatment on climacteric fruit ripening. Hortic. Res..

[51] Vilhena N.Q., Cervera-Chiner L., Moreno A., Salvador A. (2023). Recent Development in the Preharvest 1-MCP Application to Improve Postharvest Fruit Quality. New Adv. Postharvest Technol..

[52] Kou X., Feng Y., Yuan S., Zhao X., Wu C., Wang C., Xue Z. (2021). Different regulatory mechanisms of plant hormones in the ripening of climacteric and non-climacteric fruits: A review. Plant Mol. Biol..

[53] Kliewer W.M., Dokoozlian N.K. (2005). Leaf area/crop weight ratios of grapevines: Influence on fruit composition and wine quality. Am. J. Enol. Vitic..

[54] Keller M., Mills L.J., Wample R.L., Spayd S.E. (2005). Cluster thinning effects on three deficit-irrigated *Vitis vinifera* cultivars. Am. J. Enol. Vitic..

[55] Etchebarne F., Ojeda H., Hunter J.J. (2010). Leaf: Fruit ratio and vine water status effects on Grenache Noir *Vitis vinifera* L. berry composition: Water, sugar, organic acids and cations. S. Afr. J. Enol. Vitic..

[56] Botton A., Girardi F., Ruperti B., Brilli M., Tijero V., Eccher G., Populin F., Schievano E., Riello T., Munné-Bosch S. (2022). Grape Berry Responses to Sequential Flooding and Heatwave Events: A Physiological, Transcriptional, and Metabolic Overview. Plants.

[57] Peng Y., Gu X., Zhou Q., Huang J., Liu Z., Zhou Y., Zheng Y. (2022). Molecular and physiological mechanisms of advanced ripening by trunk girdling at early veraison of Summer Black’ grape. Front. Plant Sci..

[58] Clayton-Cuch D., Yu L., Shirley N., Bradley D., Bulone V., Böttcher C. (2021). Auxin Treatment Enhances Anthocyanin Production in the Non-Climacteric Sweet Cherry *Prunus avium* L.. Int. J. Mol. Sci..

[59] Liu F., Aziz R.B., Wang Y., Xuan X., Yu M., Qi Z., Chen X., Wu Q., Qu Z., Dong T. (2024). Identification of VvAGL Genes Reveals Their Network’s Involvement in the Modulation of Seed Abortion via Responding Multi-Hormone Signals in Grapevines. Int. J. Mol. Sci..

[60] Shi Y., Song B., Liang Q., Su D., Lu W., Liu Y., Li Z. (2023). Molecular regulatory events of flower and fruit abscission in horticultural plants. Hortic. Plant J..

[61] Tian Y., Xin W., Lin J., Ma J., He J., Wang X., Xu T., Tang W. (2022). Auxin coordinates achene and receptacle development during fruit initiation in *Fragaria vesca*. Front. Plant Sci..

[62] Trainotti L., Pavanello A., Casadoro G. (2005). Different ethylene receptors show an increased expression during the ripening of strawberries: Does such an increment imply a role for ethylene in the ripening of these non-climacteric fruits?. J. Exp. Bot..

[63] Lorenz D.H., Eichhorn K.W., Bleiholder H., Klose R., Meier U., Weber E. (1995). Growth Stages of the Grapevine: Phenological growth stages of the grapevine *Vitis vinifera* L. ssp. vinifera—Codes and descriptions according to the extended BBCH scale. Aust. J. Grape Wine Res..

[64] Vitali M., Tamagnone M., La Iacona T., Lovisolo C. (2013). Measurement of grapevine canopy leaf area by using an ultrasonic-based method. Oeno One.

[65] Castellarin S.D., Pfeiffer A., Sivilotti P., Degan M., Peterlunger E., Di Gaspero G. (2007). Transcriptional regulation of anthocyanin biosynthesis in ripening fruits of grapevine under seasonal water deficit. Plant Cell Environ..

[66] Simon P. (2003). Q-Gene: Processing quantitative real-time RT–PCR data. Bioinformatics.

