# Supplementary material for: The Role of Naphthaleneacetic Acid and 1-Methylcyclopropene in Preventing Preharvest Berry Dropping in Vitis vinifera L
Source: Plants (Basel). 2025 Jan 19;14(2):280. doi: 10.3390/plants14020280 (PMC11768357; doi:10.3390/plants14020280)
Supplement: Supplementary file 1 [file plants-14-00280-s001.zip › plants-3398984-supplementary.pdf]

Table S1. Seasonal trend of titratable acidity (TA; n = 300) in control (CTRL), NAA and 1-MCP treated vines. Data are expressed as mean ± SE. Different letters indicate statistically significant differences ($p < 0.05$, Student-Newman-Keuls test); no significant differences were not reported.

**Treatments**	**Titratable acidity (g L^{-1} as tartaric acid)**			
	Days after anthesis			
	**77**	**83**	**89**	**96**
**CTRL**	9.12 ± 0.05	6.20 ± 0.02 **b**	7.43 ± 0.35	7.35 ± 0.22
**NAA**	9.00 ± 0.13	6.10 ± 0.09 **b**	7.36 ± 0.45	6.46 ± 0.17
**1-MCP**	9.01 ± 0.16	8.87 ± 0.02 **a**	6.18 ± 0.06	7.30 ± 0.37

Table S2. Precipitation distribution, air temperature, relative humidity (RH) and vapour pressure deficit (VPD) from 0 to 96 DAA. Data Source: Regione Piemonte Settore Fitosanitario – Sez. Agrometeorologica.

**Date**	**DAA**	**Precipitation (mm)**	**Temperature (°C)**	**RH (%)**	**VPD (kPa)**
**12/06/2014**	**0**	0	27	51	
**13/06/2014**	**1**	0	25.4	56	
**14/06/2014**	**2**	41.<br>4	21	69	
**15/06/2014**	**3**	65.<br>8	15	95	
**16/06/2014**	**4**	1.4	17.9	77	
**17/06/2014**	**5**	6.4	15.5	84	

**18/06/2014**	**6**	0	18.2	72	
**19/06/2014**	**7**	0	21.2	58	
**20/06/2014**	**8**	0	21.1	66	
**21/06/2014**	**9**	0	21.7	72	
**22/06/2014**	**10**	0	22.4	71	
**23/06/2014**	**11**	0	22.9	73	
**24/06/2014**	**12**	0	21.5	76	
**25/06/2014**	**13**	4.4	20.8	77	
**26/06/2014**	**14**	1.6	20.1	84	
**27/06/2014**	**15**	0.2	22	76	
**28/06/2014**	**16**	0	21.4	76	

**29/06/2014**	**17**	0.6	20.9	77	
**30/06/2014**	**18**	0	22	53	
**01/07/2014**	**19**	0	20.9	63	
**02/07/2014**	**20**	0.2	20.4	77	
**03/07/2014**	**21**	0	22.3	66	
**04/07/2014**	**22**	9.2	17.4	88	
**05/07/2014**	**23**	0	20.6	71	
**06/07/2014**	**24**	0	22.6	74	
**07/07/2014**	**25**	0	21.5	77	
**08/07/2014**	**26**	5	18.9	77	

**09/07/2014**	**27**	0.2	19.3	53	
**10/07/2014**	**28**	0	19.9	59	
**11/07/2014**	**29**	0	22.5	57	
**12/07/2014**	**30**	5.6	19.6	76	
**13/07/2014**	**31**	0.8	19.8	78	
**14/07/2014**	**32**	0	22.2	67	
**15/07/2014**	**33**	0	24.4	64	
**16/07/2014**	**34**	0	24.7	67	
**17/07/2014**	**35**	0	25.9	57	
**18/07/2014**	**36**	0	26.3	59	
**19/07/2014**	**37**	0	26.2	60	

**20/07/2014**	**38**	0.8	23.4	73	
**21/07/2014**	**39**	8.6	20.5	75	
**22/07/2014**	**40**	0.8	22.4	73	
**23/07/2014**	**41**	17.<br>2	22	78	
**24/07/2014**	**42**	18.<br>8	22.3	83	
**25/07/2014**	**43**	18.<br>8	22.2	83	
**26/07/2014**	**44**	8.2	21.3	80	
**27/07/2014**	**45**	0	23	65	
**28/07/2014**	**46**	0.4	22.3	72	
**29/07/2014**	**47**	21	17.7	94	

**30/07/2014**	**48**	0	20.3	78	
**31/07/2014**	**49**	0	24	66	
**01/08/2014**	**50**	0.4	23.5	73	
**02/08/2014**	**51**	0.6	22	83	
**03/08/2014**	**52**	0.2	21.9	84	
**04/08/2014**	**53**	12.2	22.5	77	
**05/08/2014**	**54**	0	23.4	71	
**06/08/2014**	**55**	0.2	23.5	66	
**07/08/2014**	**56**	0.2	23.5	71	
**08/08/2014**	**57**	0	23.2	68	
**09/08/2014**	**58**	0.2	23.4	79	

**10/08/2014**	**59**	0.2	24.1	76	
**11/08/2014**	**60**	0	24.4	72	
**12/08/2014**	**61**	0	24.4	70	
**13/08/2014**	**62**	18.6	21.8	85	
**14/08/2014**	**63**	0.2	22.7	61	
**15/08/2014**	**64**	0	21.3	70	
**16/08/2014**	**65**	0.2	20.9	66	
**17/08/2014**	**66**	0	19.8	69	
**18/08/2014**	**67**	0	20.6	73	
**19/08/2014**	**68**	2.2	20.7	86	

**20/08/2014**	**69**	0	20.8	84	
**21/08/2014**	**70**	0	21	77	
**22/08/2014**	**71**	0	21.1	75	
**23/08/2014**	**72**	0	21.4	79	
**24/08/2014**	**73**	0	21.5	70	
**25/08/2014**	**74**	0	19.4	79	
**26/08/2014**	**75**	0	18.5	88	
**27/08/2014**	**76**	0.2	22.9	70	
**28/08/2014**	**77**	0	22	78	
**29/08/2014**	**78**	0	22.2	78	
**30/08/2014**	**79**	0	23.2	70	

**31/08/2014**	**80**	0	22.8	73	
**01/09/2014**	**81**	0	21.6	53	
**02/09/2014**	**82**	0	20.3	50	
**03/09/2014**	**83**	0	18.5	64	
**04/09/2014**	**84**	0	22.4	57	
**05/09/2014**	**85**	0	20.9	67	
**06/09/2014**	**86**	0	21.9	67	
**07/09/2014**	**87**	0	23.2	63	
**08/09/2014**	**88**	0	23.5	68	
**09/09/2014**	**89**	29.2	21.8	78	

**10/09/2014**	**90**	2.8	20.8	85	
**11/09/2014**	**91**	13.<br>2	21.1	82	
**12/09/2014**	**92**	0	20.1	68	
**13/09/2014**	**93**	0.2	19.5	68	
**14/09/2014**	**94**	0	19.8	76	
**15/09/2014**	**95**	7.8	20.1	81	
**16/09/2014**	**96**	0	20.4	78	

Table S3. Primers for genes used in the research.

Gene ID	FORWARD	REVERSE
VIT_212s0059g0138<br>0	GACTCCGAGCCCACACTGTGCCG	GGAGGCCGCTGACCGTGTCGTCTTG
VIT_211s0016g0238<br>0	TTGGTTTGGAGAAGGGCTACATAAGAGA<br>A	GCCTGGAACTTGATCATCTTGGAGCA
VIT_207s0005g0082<br>0	ATCCTCTATTTGTCAGCCCTATACCCCA	GGGTACACAGACTCCGAGTCAGAGCC<br>G

VIT_216s0013g00980	CTGGCCGGAAAAGGGAGAGAGACAGTG	CCCCTAAAACATGACTGGCTGCCGC
VIT_203s0091g00310	GCTGAACTCAGTGTACCGACAAGGG	CTGGTTAATGGAGGCTCCTCTGGA
VIT_207s0104g00800	CAGAAGTGGAGTTTGTGATGAGGGCC	ACTCTGTAACATCTGGCATTTTCACA
VIT_219s0015g01190	CTATATGCTCGCTGCTGACG	AAGCCAGGCAGAGACAACTC
